# Multicopy Chromosome Integration and Deletion of Negative Global Regulators Significantly Increased the Heterologous Production of Aborycin in *Streptomyces coelicolor*

**DOI:** 10.3390/md21100534

**Published:** 2023-10-13

**Authors:** Jia-Yi Li, Jun-Yu Liang, Zhao-Yuan Liu, Yue-Zhao Yi, Jing Zhao, Zhi-Yong Huang, Jun Chen

**Affiliations:** 1 Department of Marine Biological Science & Technology, College of Ocean and Earth Sciences, Xiamen University, Xiamen 361102, China; 22320191151003@stu.xmu.edu.cn (J.-Y.L.); y__vette@sina.com (J.-Y.L.); liuzhaoyuan2919@163.com (Z.-Y.L.); 22320182201398@stu.xmu.edu.cn (Y.-Z.Y.); sunnyzhaoj@xmu.edu.cn (J.Z.); 2 State-Province Joint Engineering Laboratory of Marine Bioproducts and Technology, Xiamen University, Xiamen 361102, China; 3 Xiamen City Key Laboratory of Urban Sea Ecological Conservation and Restoration, Xiamen University, Xiamen 361102, China; 4 Tianjin Institute of Industrial Biotechnology, Chinese Academy of Sciences, Tianjin 300308, China; 5 National Technology Innovation Center of Synthetic Biology, Tianjin 300308, China

**Keywords:** RiPP, marine *Streptomyces*, *phoU* (*SCO4228*), *wblA* (*SCO3579*), *SCO1712*, *orrA* (*SCO3008*), *gntR* (*SCO1678*)

## Abstract

Aborycin is a type I lasso peptide with a stable interlocked structure, offering a favorable framework for drug development. The aborycin biosynthetic gene cluster *gul* from marine sponge-associated *Streptomyces* sp. HNS054 was cloned and integrated into the chromosome of *S. coelicolor* hosts with different copies. The three-copy *gul*-integration strain *S. coelicolor* M1346::3*gul* showed superior production compared to the one-copy or two-copy *gul*-integration strains, and the total titer reached approximately 10.4 mg/L, i.e., 2.1 times that of the native strain. Then, five regulatory genes, *phoU* (*SCO4228*), *wblA* (*SCO3579*), *SCO1712*, *orrA* (*SCO3008*) and *gntR* (*SCO1678*), which reportedly have negative effects on secondary metabolism, were further knocked out from the M1346::3*gul* genome by CRISPR/Cas9 technology. While the *ΔSCO1712* mutant showed a significant decrease (4.6 mg/L) and the *ΔphoU* mutant showed no significant improvement (12.1 mg/L) in aborycin production, the *ΔwblA*, *ΔorrA* and *ΔgntR* mutations significantly improved the aborycin titers to approximately 23.6 mg/L, 56.3 mg/L and 48.2 mg/L, respectively, which were among the highest heterologous yields for lasso peptides in both *Escherichia coli* systems and *Streptomyces* systems. Thus, this study provides important clues for future studies on enhancing antibiotic production in *Streptomyces* systems.

## 1. Introduction

Aborycin is a class I lasso peptide and was first isolated from *Streptomyces* sp. SP9440 as a novel anti-HIV metabolite with the origin name RP 71,955 [1,2]. It was independently rediscovered from soil *S. griseoflavus* Tü 4072 as an antibiotic [3], then from deep-sea *Streptomyces* sp. SCSIO ZS0098 as an anti-infective natural product [4] and from marine sponge-associated *Streptomyces* sp. MG010 as an antibacterial marker for screening gain-of-function mutants [5]. It was also predicted to be expressed in the mangrove soil strain *Streptomyces* sp. EMB24 [6]. Aborycin has a typical lasso topology in which the N-terminal 9 amino acids form a macrocyclic ring, and the C-terminal 12 amino acids form a tail that folds back and threads through the ring [2]. Two disulfide bonds between the ring and the tail further increase the structural stability and distinguish class I lasso peptides from others [7,8].

Lasso peptides are a growing class of bioactive bacterial peptides with unique lasso topology, which differentiates them from other members within the much larger ribosomally synthesized and post-translationally modified peptide (RiPP) superfamily [9,10]. The compact and constrained topology endows most lasso peptides with remarkable thermal and proteolytic stability and favors peptide–protein interactions, accounting for the diverse biological activities of lasso peptides, mainly as enzyme inhibitors and receptor antagonists [7,11]. Robust scaffolds of lasso peptides have attracted attention towards drug development, such as epitope grafting [12], the incorporation of noncanonical amino acids [13] and protein fusion [14]. While these modifications provide the opportunity to develop novel biological activities with therapeutic potential, they always lead to lower production levels [15]. Moreover, genome mining approaches have greatly accelerated lasso peptide discovery in recent years. Since the first lasso peptide was isolated by genome mining in 2008 [16], the number of lasso peptides discovered by such approaches has steadily increased [11]. By applying the RODEO algorithm, >1400 lasso peptide biosynthetic gene clusters (BGCs) were identified from DNA sequence databases, a great increase over the previously known numbers [17]. Although lasso peptide BGCs are widely distributed among bacteria, only approximately 80 lasso peptides were previously characterized [18]. Therefore, both drug development and functional characterization require effective production systems to explore this rich source of lasso peptides and their modifications.

The heterologous production of lasso peptides in *E. coli* often provides higher yields than the use of native producers. However, this production advantage in *E. coli* seems to be confined to lasso peptides from Proteobacteria and is less viable for clusters from other phyla [11,15]. Specially, inefficiency in expressing genes with high GC content, the lack of metabolic precursors and the lack of detoxification mechanisms are the main weakness of the *E. coli* system in terms of expressing natural products from actinomycetes [19,20]. Recently, *Streptomyces* hosts, especially *S. coelicolor*, *S. lividans* and *S. albus*, have shown significant potential for producing lasso peptides from actinobacteria [17,21,22]. Several technological advances have also been achieved for *Streptomyces* systems, such as CRISPR/Cas9 genome editing technology [23,24] and the multiplexed site-specific genome engineering (MSGE) method [25]. Genome editing offers a rapid way to modify regulatory elements involving secondary metabolism and therefore impact heterologous production. The MSGE method led to the successful development of a panel of *S. coelicolor* heterologous hosts, in which up to five copies of BGCs could be integrated into the specific sites of the host chromosome in a single step, leading to significant yield improvements [25]. In our previous study, an aborycin BGC was identified from a marine sponge-associated *Streptomyces* sp. HNS054 [5]. This provided an opportunity to produce this lasso peptide heterologously in *Streptomyces* systems. Thus, in this study, a *Streptomyces* system for aborycin production that is compatible with up-to-date technologies was established. By increasing the integrated copy numbers of the aborycin BGC in the host chromosome and by deleting the negative global regulatory genes involved in secondary metabolism by genome editing, the production of aborycin was significantly improved. This study provides a useful reference to improve *Streptomyces* systems for the production of lasso peptides and their modifications and thus benefits drug development and functional characterization.

## 2. Results

### 2.1. General Information on the Aborycin Gene Cluster from Streptomyces sp. HNS054

**Figure 1:**
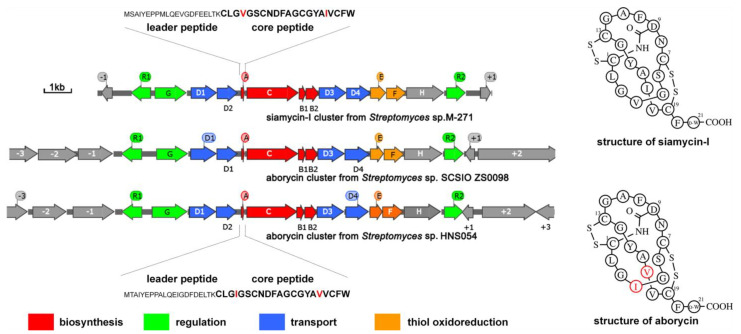
General information on aborycin and siamycin-I and their BGCs, sequences and secondary structures. Sequence differences between aborycin and siamycin-I are marked in red.

As previously reported [5], the aborycin gene cluster *gul* (named after Gulei Town, where the host marine sponge was collected from its coastal sea) from marine sponge-associated *Streptomyces* sp. HNS054 consists of 14 ORFs spanning an approximately 12 kb region from *gulR1* to *gulR2* (Figure 1). Sequence alignment showed that the DNA sequence of this region shares 98% identity to the aborycin gene cluster *abo* from *Streptomyces* sp. SCSIO ZS0098 [4]. The *gul* cluster also shows high identity to the siamycin-I gene cluster *msl* from *Streptomyces* sp. M-271 [26] in gene sequence and gene organization (Figure 1). *GulA* encodes a 42-residue peptide with a leader peptide at its N-terminus and a 21-residue core peptide (CLGIGSCNDFAGCGYAVVCFW) at its C-terminus. The primary and secondary structures of aborycin and siamycin-I are almost the same, except that the residues at the 4th and 17th positions are switched (Figure 1). Due to the high similarity between aborycin and siamycin-I, commercial reagents of siamycin-I could be used as a reference to locate the high-performance liquid chromatography (HPLC) signal of aborycin.

### 2.2. Cloning of the Gul BGC and Construction of Strains for Heterologous Expression of Aborycin

The cloning procedure of the *gul* BGC is shown in Figure S1. The 14 kb fragment containing the *gul* BGC was amplified from Streptomyces sp. HNS054 by high-fidelity PCR and subsequently cloned and inserted into the integrative vector pSAT209, yielding pSAT-GUL (Figure S2). The plasmid was fully sequenced, and the results confirmed that no mutation occurred within the plasmid frame nor the *gul* ORFs. Then, the plasmid pSAT-GUL was transferred to *S. coelicolor* strains M1146, M1246, M1346, M1446 and M1546 by conjugation. As a blank control, the empty plasmid pSAT209 was also transferred to *S. coelicolor* M1146. The numbers of successful *attP*-*attB* recombinations in exconjugants were determined by PCR. The results showed that strains M1146::*gul* (Figure S3A), M1246::2*gul* (Figure S3B) and M1346::3*gul* (Figure S3C) were successfully constructed. However, four-copy and five-copy integrations were unsuccessful after four rounds of conjugation and screening.

### 2.3. Extraction and Detection of Aborycin

**Figure 2:**
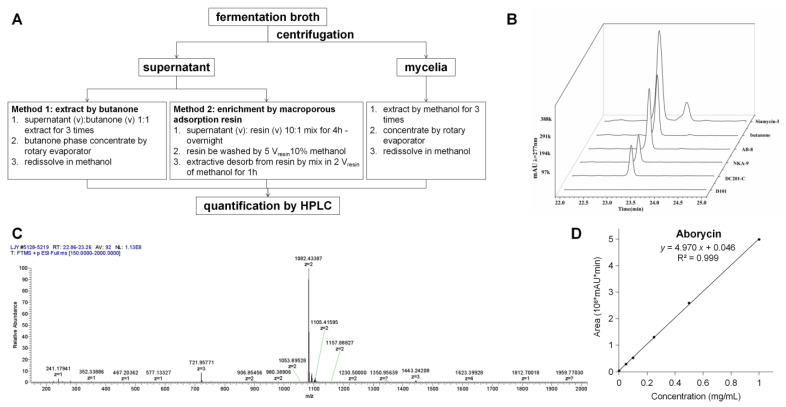
Extraction of aborycin. (**A**). The extraction procedure. (**B**). The 23.2 min HPLC peaks of extractives obtained from the supernatant of M1346::3*gul* by butanone or by macroporous adsorption resins. Siamycin-I was used as a control. The loading quantities of each test were adjusted to represent the same amount of the supernatant. (**C**). Mass spectrum analysis of the matter abundance in the *m*/*z* range [150–2000] of the 23.2 min collection from the sample enriched by the AB-8 resin. A peak at *m*/*z* = 1082.43 (*z* = 2) accounted for approximately 90% of the total mass. (**D**). The standard curve of concentrations of the HPLC peak areas of aborycin.

With siamycin-I as the control, the M1346::3*gul* strain was used to test whether aborycin was successfully produced. Aborycin was extracted, as shown in Figure 2A. The butanone extract had an HPLC signal at 23.2 min, which was the same as the signal of siamycin-I (Figure 2B). In the following test of the macroporous adsorption resin method, the AB-8 resin showed high enrichment efficiency for aborycin that was equivalent to that of the butanone method. Mass spectrum analysis confirmed that a substance with a relative molecular mass of m/z = 1082.43 (z = 2) accounted for the majority of the composition at the 23.2 min peak of the AB-8-enriched samples (Figure 2C). The MS data of this substance were perfectly matched to the aborycin data from a previous report [5]. Thus, two facts were confirmed by these tests: First, the M1346::3*gul* strain can express aborycin. Second, compared with the butanone method, the AB-8 resin method offered a safer, easier and cheaper method to enrich aborycin from the fermentation supernatant. This method was thus applied in the following quantification studies: After pure aborycin was isolated from 5.6 L of culture broth containing the M1346::3*gul* strain, a precise standard curve of concentrations to the HPLC peak areas of aborycin was obtained (Figure 2D). By applying this curve, aborycin production from different strains or conditions could be quickly quantitated by HPLC. The ^1^H NMR spectral data of aborycin were also obtained to confirm the structure (Figure S4).

### 2.4. Yield Comparison among Different Strains

**Figure 3:**
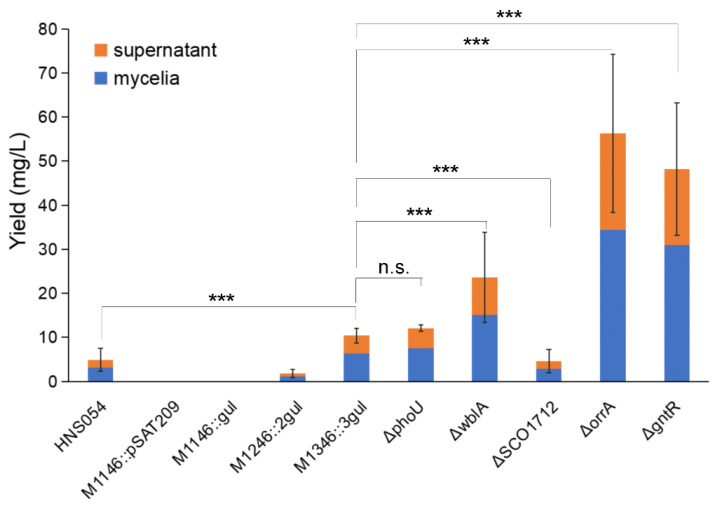
Aborycin titers from different strains. HNS054, the wild-type strain *Streptomyces* sp. HNS054. *ΔphoU*—*ΔgntR*, gene knockout strains from *S. coelicolor* M1346::3*gul*. Orange block, average production from supernatant extracted by the AB-8 method. Blue block, average production from mycelia extracted by the methanol method. Error bar, standard deviation of total titers (supernatant + mycelia). Data were counted from triplicate experiments. ***, *p* < 0.001. n.s., *p* > 0.05.

Titers of aborycin from different strains are shown in Figure 3. Adding the supernatant and mycelial products, the native HNS054 strain average yielded 4.9 ± 2.6 mg/L aborycin. No aborycin signal was detected from the blank control M1146::pSAT209 and the one-copy strain M1146::*gul*. Signals became obvious for the M1246::2*gul* and M1346::3*gul* strains, which had average titers of 1.8 ± 1.0 mg/L and 10.4 ± 1.6 mg/L, i.e., 0.4 times and 2.1 times that of the native strain, respectively. Both supernatant and mycelia contributed to the production, accounting for approximately 38 percent and 62 percent, respectively.

Then, the M1346::3*gul* strain was selected for genetic modification to verify whether aborycin production could be further improved by deleting negative regulatory genes (Table 1), which were reported to act at higher levels of the gene-regulatory networks to control secondary metabolism, whose knockout mutants always resulted in an increase in secondary metabolism. The *ΔphoU*, *ΔwblA*, *ΔSCO1712*, *ΔorrA* and *ΔgntR* mutants were successfully constructed (Figures S5 and S6). A titer study showed that the aborycin production of the M1346::3*gul ΔphoU* strain was 12.1 ± 0.7 mg/L, which was slightly higher than that of the M1346::3*gul* strain but not significantly different (*p* > 0.05). The *ΔwblA* strain was significantly (*p* < 0.01) higher than the M1346::3*gul* strain, with a titer of 23.6 ± 10.2 mg/L, i.e., 4.8 times that of the native strain or 2.3 times that of the strain before *wblA* knockout. Greater improvements were displayed in the *ΔorrA* and *ΔgntR* mutants, which had titers of 56.3 ± 18.0 and 48.2 ± 15.1 mg/L, respectively (Figure 3).

## 3. Discussion

In this study, efforts were made to use the *Streptomyces* system for the heterologous production of aborycin. First, the cloning procedure was simplified. The 14 kb genomic fragment containing the *gul* gene cluster was amplified by high-fidelity PCR, and sequencing confirmed its accuracy. High-fidelity PCR techniques have been developed to amplify long DNA fragments with lengths as long as 15–20 kb. The cloning of RiPP gene clusters could benefit from these developments because a large portion of RiPP gene clusters have lengths below 15 kb (Table S3). In a recent review on newly discovered RiPPs, out of the 33 listed gene clusters, 25 (76%) had lengths below 15 kb ([32], Table S4). Second, to facilitate foreign DNA integration following genome editing, the integrative vector pSET152 was modified to pSAT209 to prevent challenges related to antibiotic resistance. Third, the extraction of aborycin from the supernatant was optimized. Aborycin is a peptide with amphiphilic characteristics and thus has reversible affinity to certain macroporous adsorption resins. In this case, AB-8 resin was found to be such a resin, adsorbing aborycin in a water solution and desorbing it in methanol. The optimal AB-8 resin method condition is that the supernatant and AB-8 resin are 10:1 (*v*/*v*) mixed for 4 h to overnight, and then the resin is washed with a 5 × resin volume of 10% methanol following the desorption of the aborycin from the resin by a 2 × resin volume of methanol for 1 h. Compared with the butanone method [4,5], the AB-8 resin method recovered a majority of the aborycin from the supernatant with minimal organic solvent and minimal labor. The desorbed extract is also much cleaner than the butanone extract. By simply adsorbing and desorbing, approximately 40% of the total production was recovered from the culture supernatant. A similar situation occurred with the heterologous expression of sviceucin, where approximately 1/3 of the product was released in the culture supernatant [21]. Large-scale production would discard the supernatant because of the high cost of solvent extraction. With this optimization, the product in the supernatant could be recovered at a low cost. We speculated that this theory and operation could be applied to other RiPPs due to their peptide nature. Finally, the multiple chromosomal integration of foreign gene clusters following CRISPR/Cas9 genome editing was successfully achieved, and the best case resulted in a 25-fold increase in aborycin production compared to the native strain.

Multiple chromosomal integration of foreign BGC was proven to be a mature technique to increase heterologous expression. The approach was successfully demonstrated in *S. coelicolor* [25], *S. albus* J1074 [33] and *S. lividans* [34]. In this study, one-copy integration showed no signal, while three-copy integration showed a 2.1-fold increase compared to the native strain. However, more advanced techniques were needed to obtain four- or five-copy integration in one single step. In our attempts to transfer other BGCs into these M1146-M1546 hosts, the number of exconjugants decreased rapidly with increasing integrated copy number. It was previously speculated that a high copy number of chromosomal integration caused the accumulation of target products, endangering bacterial growth [25]. However, the titer of aborycin was further improved 5.4-fold by *orrA* gene knockout, implying that the *Streptomycete* actually tolerates higher concentrations of aborycin.

CRISPR/Cas-based genome editing tools provide swift, accurate and traceless ways to modify the genomes of S*treptomyces* [35]. A straightforward way to exploit the CRISPR/Cas9 tools was to delete negative regulator genes. This study proposed deleting genes that were reported to have a negative impact on secondary metabolism at a high level of the regulation networks. Five mutants, *ΔphoU*, *ΔwblA*, *ΔSCO1712*, *ΔorrA* and *ΔgntR,* were successfully constructed from the M1346::3*gul* strain.

**Figure 4:**
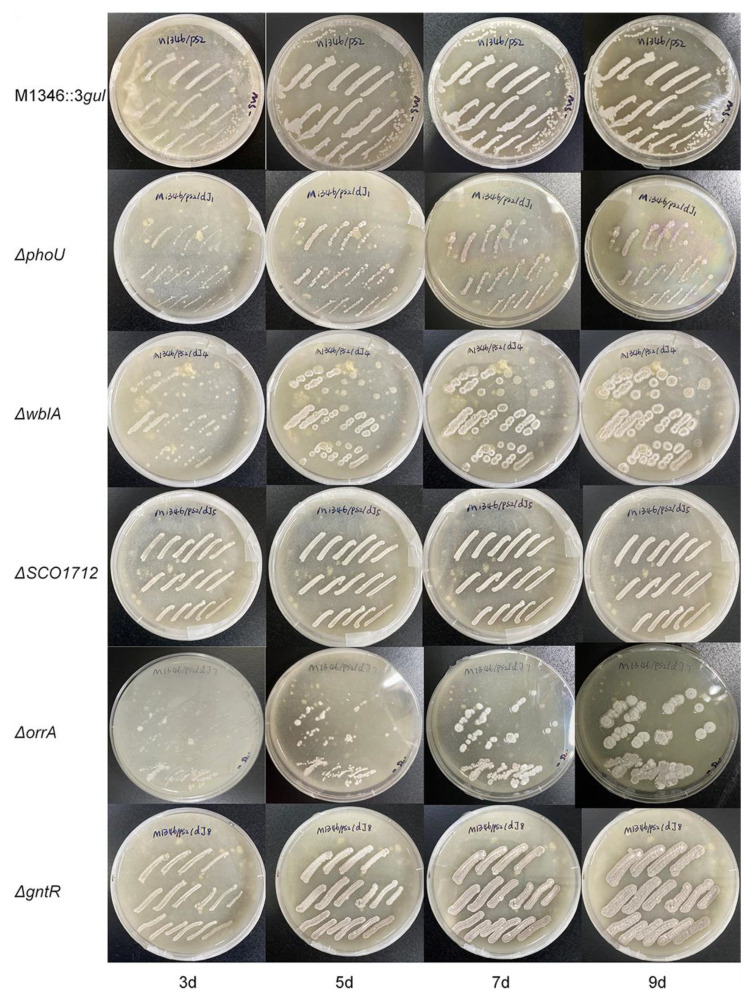
The growth of strains on MS-agar plates from 3 days to 9 days. *ΔphoU*—*ΔgntR*, gene knockout strains from *S. coelicolor* M1346::3*gul*.

Although the detailed function of the *phoU* gene was unclear, it was speculated to be involved in the pho regulon, which responds to phosphate starvation. PhoR senses such conditions, and then PhoP is phosphorylated following PhoP-P binding to specific sequences named PHO boxes, thus activating or repressing a set of genes [36]. Under phosphate starvation conditions, the *ΔphoU* mutants showed an approximately six-fold increase in the production of actinorhodin [27]. Our study showed that aborycin production in the *ΔphoU* mutants was not significantly improved from that in the strain before mutation when strains were cultured in R5 medium. Moreover, the growth of the *ΔphoU* mutants on MS-agar was retarded in a 9-day morphology observation (Figure 4). This type of the mutant requires more optimization before it can be applied in antibiotic production.

The *wblA* gene was reported as a pleiotropic downregulator of antibiotic biosynthesis in *S. coelicolor*. *ΔwblA* mutants exhibited a defect in sporulation, achieved higher biomass than the wild type, and overproduced secondary metabolites [37]. Overproduction of antibiotics by disruption of the *wblA* orthologs was also observed in other *Streptomyces* bacteria [38]. This study confirmed that the production of aborycin in the *ΔwblA* mutant was significantly improved by two-fold. Higher growth and defects in sporulation were also observed (Figure 4).

SCO1712 is a member of the TetR family. TetR family transcriptional regulators are among the most common prokaryotic transcriptional regulators. When *SCO1712* was overexpressed or disrupted, ACT production decreased or increased compared with that in *S. coelicolor* M145, respectively, suggesting that *SCO1712* is a pleiotropic downregulator of antibiotic biosynthesis in *S. coelicolor* [29]. It was further speculated that there is a synergistic effect between *SCO1712* and precursor flux pathways in antibiotic production [39]. Unfortunately, in this study, after *SCO1712* knockout, the production of aborycin decreased significantly. This provides ideas for future optimizations of precursor flux pathways or mediums for this mutant.

*OhkA* (*SCO1596*)—*OrrA* (*SCO3008*) is a group of prokaryotic two-component regulatory systems with highly similar transcriptomic features. *ΔorrA* mutants lead to the significant overproduction of antibiotics and the downregulation of the *bld*, *chp*, *rdl*, and *wbl* genes associated with morphological development [30]. Correspondingly, we found that *ΔorrA* mutants overproduced approximately 4.4-fold more aborycin than the strains before mutation (Figure 3). Their morphological development was also similar to that of the *ΔwblA* mutants (Figure 4). These results implied that the *OrrA* regulatory system likely covered the *wbl* regulatory system and controlled a wider range of resources for antibiotic production. 

The bacterial GntR family is one of the most abundant groups of helix-turn-helix transcription factors that respond appropriately to metabolite microenvironments [40]. It was reported that deletion of a GntR-like gene allowed for platensimycin and platencin overproduction in *S. platensis* [41]. *GntR* (*SCO1678*) of *S. coelicolor* encodes a repressor protein to control the gluconate operon, which enables *Streptomyces* to utilize gluconate in the media [31]. No obvious evidence has linked *GntR* (*SCO1678*) to antibiotic overproduction to date. Interestingly, both the overproduction of aborycin (Figure 3) and overgrowth (Figure 4) were observed from the *ΔGntR* mutants in this study. No additional gluconate was added to the R5 or MS-agar media to obtain these results. These observations provide a significant gene that is worthy of further study to discover underlying metabolic regulatory mechanisms.

To further improve the production of aborycin, we attempted to double knockout the genes *orrA* and *gntR*. It was difficult to obtain enough spores from the *ΔorrA* strains. Thus, we tried to knock out the *orrA* gene from the M1346::*3gul ΔgntR* strain. The cloning process was difficult, and positive clones showed contradictory yields. We reason it in part to genetic instability, which might result from unknown genetic changes by one round of multi-sites of *attP*/*attB* recombination and two rounds of Cas9-based CRISPR. Thus, the cloning strategy should be carefully planned to achieve double knockout or triple knockout. It could be feasible that before the integration of the heterogeneous BGCs, stable *S. coelicolor* M1346-based double-knockout or triple-knockout hosts should be constructed by one round of multiplex genome editing [23,25] to reduce genetic instability risks, which might be brought from two or more rounds of CRISPR.

Although great production was achieved by these genetic modifications, we believe the *Streptomyces* systems could be further improved. With more global regulators that govern secondary metabolism being characterized [42], it is worth manipulating them one by one to obtain further knowledge.

## 4. Materials and Methods

### 4.1. Strains, Plasmids and Primers

The strains and plasmids used in this study are listed in Table S1, and the primers used in this study are listed in Table S2. The construction of the related vectors is shown in Figures S1 and S2.

### 4.2. Construction of Heterologous Expression Strains to Produce Aborycin

Foreign DNA integration could be fulfilled by the integrative plasmid pSET152 [43], and genome editing could be executed by the suicide plasmid pKCcas9 [24]. However, both vectors contain the apramycin resistance gene *aac(3)IV*. To achieve BGC integration following genome editing, the antibiotic-resistance markers between the two genetic manipulations should be different. Thus, the *aac(3)IV* gene in pSET152 was replaced by the ampicillin resistance gene *bla* from the pUC57 plasmid and the thiostrepton resistance gene *tsr* from the pGM1190 plasmid. The resulting plasmid was named pSAT209 (Figure S1). The Genomic DNA of *Streptomyces* sp. HNS054 was extracted from the later logarithmic phase cells using a bacterial genomic DNA extraction kit (Cat # DP2001, Biotake, Beijing, China). Using primers 054LasF and 054LasR, a 14 kb DNA fragment containing the *gul* BGC (located at 15932–29864 nt of GenBank: AC003_RS35325) was amplified from the *Streptomyces* sp. HNS054 genome (Assembly: GCF_001044185.1). After gel purification, the *gul* fragment was assembled with *Eco*RV-linearized pSAT209 using a one-step cloning kit (Cat # C113, Vazyme, Nanjing, China). The resulting vector pSAT-GUL (Figure S1) was sequenced to confirm the accuracy. Then, it and the control plasmid pSAT209 were transferred into *E. coli* ET12567/pUZ8002 competent cells and conjugated into *S. coelicolor* M1146-M1546 hosts by standard protocols [44]. Exconjugants were randomly picked to check *attP-attB* recombination by PCR with the following primers: The forward primer ID-oriT-fw targets a position approximately 400 bp upstream of the attP locus in the vector pSAT-GUL. The reverse primers, ID-native-attB-Rev, ID-CPK-attB-Rev, ID-RED-attB-Rev, ID-CDA-attB-Rev and ID-ACT-attB-Rev, each target a position downstream of an individual *attB* locus in the *S. coelicolor* M1546 genome. The integrated copy number of the *gul* gene cluster in the host genome was determined by the number of positive *attP-attB* recombinations [25].

### 4.3. Gene Knockout in S. coelicolor M1346::3gul by the CRISPR/Cas9 Method

The *phoU* (*SCO4228*), *wblA* (*SCO3579*), *SCO1712*, *orrA* (*SCO3008*) and *gntR* (*SCO1678*) genes (Table 1) in *S. coelicolor* M1346::3*gul* were independently knocked out by the CRISPR/Cas9 genome editing method [24]. To generate the *ΔphoU* mutant, the sgRNA expression cassette (f1 fragment) was PCR amplified with the plasmid pKCcas9dO as the template and f1J01-fwd/f1gRNA-R as primers. The upstream (f2 fragment) and downstream (f3 fragment) regions of the *phoU* gene were PCR amplified with the primer pairs f2J01-fwd/rev and f3J01-fwd/rev, respectively. Then, three DNA fragments were assembled by overlapping PCR using primers f1J01-fwd and f3J01-rev. The resulting DNA fragment was cloned and inserted into pKCCas9 using a one-step cloning kit (Cat # C113, Vazyme, Nanjing, China) to yield pKCCas9-dJ01. The obtained plasmid was introduced into the M1346::3*gul* strain by conjugal transfer. The correct knockouts were verified by PCR using primers ID-dJ01-fwd/rev. By using the J04, J05, J07 and J08 sets of primers and the same procedure, pKCCas9-dJ04, -dJ05, -dJ07 and -dJ08 were generated and yielded the *ΔwblA*, *ΔSCO1712*, *ΔorrA* and *ΔgntR* mutants after successful conjugation into the M1346::3*gul* strain, respectively. The positions of the spacers, primers and target genes are shown in Figure S5.

### 4.4. Metabolite Analysis

The wild-type *Streptomyces* sp. HNS054 and the *S. coelicolor* strains were grown on MS solid medium to achieve sporulation. Approximately 10^8^ spores (or 40 μL mycelia store) were transferred to 40 mL R5 medium with appropriate antibiotics and cultured at 200 rpm and 28 °C for 3 days as the seed solution. Ten milliliters of the seed was transferred to 500 mL of R5 medium and cultured at 200 rpm and 28 °C for 9 days to complete the fermentation. Aborycin was extracted, as shown in Figure 2A. The fermented broth was centrifuged to separate the supernatant from the mycelia and then independently extracted. The supernatant was extracted by two methods. The first method was butanone extraction three times, and then the butanone phase was concentrated by rotary evaporation. Then, four macroporous adsorption resins, AB-8, DC201-C, NK-9 and D101 (Hecheng New Material, Zhengzhou, China), were tested for their aborycin recovery efficiencies. The AB-8 resin had a high enrichment ratio for aborycin (Figure 2B); therefore, AB-8 was used as the second method to extract aborycin. Because the AB-8 resin method was safer, easier and cheaper, all aborycin titers of the supernatants in this study were obtained by this method. The mycelium was extracted three times with 300 mL methanol and then concentrated by rotary evaporation. Extracts from both the supernatant and mycelia were adjusted to a final concentration of approximately 5 mg/mL in methanol and filtered with 0.22 µm filter membranes before HPLC or LC—MS analysis. Analytical HPLC was performed using a Shimadzu Prominence LC-20A (Shimadzu, Tokyo, Japan) with a 2.6 × 250 mm Ultimate XB-C18 column (Welch, Shanghai, China). The elution conditions were 1% solvent B for 0–7 min and then a linear gradient to 95% solvent B (solvent B: 0.1% formic acid in CH_3_CN; solvent A: 0.1% formic acid in H_2_O) for 7–30 min at a flow rate of 1 mL/min and monitored at 277 nm. To locate the aborycin signal, the commercial siamycin-I reagent (Adipogen Life Sciences, San Diego, CA, USA) was used as the control. Because the amino acid sequences between aborycin and siamycin-I were the same except the switched residues at the 4th and 17th positions (Figure 1), it is reasonable to expect the HPLC signal position of aborycin to be slightly different from that of siamycin-I. LC—MS analysis was performed using a Q Exactive spectrometer (Thermo Scientific, Waltham, MA, USA) with the same elution program as HPLC. The software MZmine 2 [45] was used to assess the chromatograms.

### 4.5. Purification of Aborycin, the Calibration Curve and ^1^H-NMR Spectra

A 5.6 L culture broth of the M1346::3*gul* strain was prepared as described in the previous section. The crude extract was enriched by the AB-8 resin method from the supernatant and then purified by semipreparative HPLC with a YMC-PACK ODS-A column (10 × 250 mm, φ 5 μm, YMC, Kyoto, Japan). F1–F4 fractions near the target position were collected, and the F2 fraction was determined by analytical HPLC to be the major fraction containing aborycin. Then, approximately 22 mg of the F2 fraction was obtained after rotary evaporation. Ten milligrams of the F2 fraction was dissolved in 2 mL of methanol, and analytical HPLC was run as described in the previous section. The 23.2 min peaks were collected and concentrated to approximately 4 mg of pure aborycin in the form of a white powder. A methanol solution of aborycin was prepared with a precise concentration of 1.00 mg/mL and then diluted to a series of concentrations. These dilutions were subjected to analytical HPLC, and the 23.2 min peak areas were recorded. A standard curve of concentrations to HPLC peak areas of aborycin was then constructed (Figure 2D), and the following formula was deduced.

y = 4.970 x + 0.046 (R^2^ = 0.999) (1)

where y (10^6^∙mAU∙min) is the peak area of the HPLC signal of aborycin, and x (mg/mL) is the concentration.

Approximately 3.5 mg of aborycin was dissolved in 500 μL DMSO, and the ^1^H NMR (600 MHz) spectra of aborycin were obtained using a Bruker Avance III HD 600 MHz spectrometer (Bruker, Billerica, MA, USA).

## 5. Conclusions

This study demonstrated improvements in the *S. coelicolor* system for aborycin overproduction. The maximum shake flask titer was over 50 mg/L, which is comparable to the best examples of *E. coli* systems or *Streptomyces* systems for lasso peptide heterologous expression [15]. We confirmed that increasing the copy number of chromosome integration and disrupting the negative global regulators governing secondary metabolism were effective ways to improve antibiotic production in *S. coelicolor* systems. *GntR* (*SCO1678*) was proposed to be a significant target that is worthy of further studies on metabolic regulatory mechanisms. The optimized extraction method, which could efficiently recover aborycin from the supernatant, also contributed to the total titer. Due to the similar chemical properties of RiPPs to aborycin, this tactic is also suitable for the development of other RiPP relatives. By applying these modifications to *Streptomyces* systems, the drug development and functional characterization of new lasso peptides from genome mining will be greatly improved.

## Figures and Tables

**Table 1:** Information on the genes to be knocked out.

Trial	Gene Code	Symbol	Relevant Features	References
J01	*SCO4228*	*phoU*	*ΔphoU* mutant showed 6-fold increase in ACT production when phosphate starvation	[27]
J04	*SCO3579*	*wblA*	*ΔwblA* mutant showed 1.5-fold increase in doxorubicin production	[28]
J05	*SCO1712*	*SCO1712*	*ΔSCO1712* mutant showed 1.62-fold or 1.22-fold increase in ACT or RED production, respectively	[29]
J07	*SCO3008*	*orrA*	*ΔorrA* mutant showed great increase in ACT and RED production	[30]
J08	*SCO1678*	*gntR*	*ΔgntR* mutant altered the secondary metabolite profile of *S. coelicolor*	[31]

## Data Availability

Not applicable.

## Supplementary Materials

The following supporting information can be downloaded at https://www.mdpi.com/article/10.3390/md21100534/s1, Figure S1: Construction of the integrative vector for aborycin expression; Figure S2: Cloning of the *gul* gene cluster; Figure S3: PCR validation of *gul* cluster integration in the *attB* loci. Figure S4: ^1^H NMR (600 MHz) spectrum of aborycin in DMSO. Figure S5: CRISPR/Cas9 constructions of the five gene-knockout mutants from M1346::3*gul*; Figure S6: PCR screening for positive mutants; Table S1: Information of plasmids and strains; Table S2: Primer sequence used in this study; Table S3: The length of RiPP biosynthetic gene clusters; Table S4: RiPP examples reported by Montalbán-López et al [46,47,48,49,50,51,52,53,54,55,56].
