# Supplementary material for: Multicopy Chromosome Integration and Deletion of Negative Global Regulators Significantly Increased the Heterologous Production of Aborycin in Streptomyces coelicolor
Source: Mar Drugs. 2023 Oct 13;21(10):534. doi: 10.3390/md21100534 (PMC10608281; doi:10.3390/md21100534)
Supplement: Supplementary file 1 [file marinedrugs-21-00534-s001.zip › marinedrugs-2636467-supplementary.pdf]

Multicopy Chromosome Integration and Deletion of Negative Global Regulators Significantly Increased the Heterologous Production of Aborycin in *Streptomyces coelicolor*

Supplementary Figures and Tables

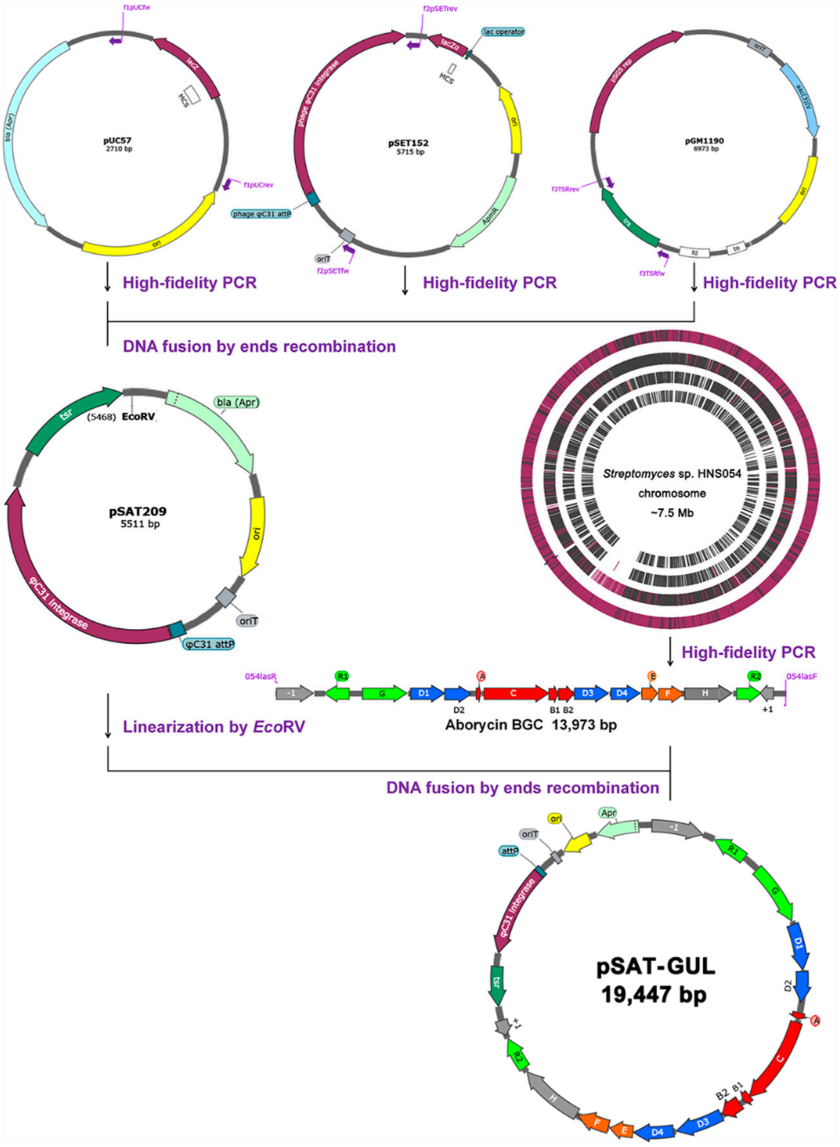

Figure S1 Construction of the integrative vector for aborycin expression. The f1 fragment (*bla*+*ori*^{pUC}) was amplified from the pUC57 plasmid by primers f1pUCfw and f1pUCrev. The f2 fragment (*oriT*+*attP*+*int*) was amplified from the pSET152 plasmid by primers f2pSETfw and f2pSETRev. And the f3 fragment (*tsr*) was amplified from the pGM1190 plasmid by primers f3TSRfw and f3TSRrevf2. Then, the three fragments were fused into the integrated plasmid

pSAT209 by using a Multi-fragment Assembly Kit (CAT # C113, Vazyme, Nanjing, China). By using the same assembly kit, the *Eco*RV-linearized pSAT209 was fused with the 14 Kb aborycin BGC fragment, which was amplified from the genomic DNA of the HNS054 strain with primers 054LasF and 054LasR, to form the final integrative vector pSAT-GUL. Colonies were screened by PCR with primers gul-cF and gul-cR. The positive pSAT-GUL plasmid was whole-sequenced to confirm sequence accuracy. All high-fidelity PCR reactions were performed by using a DNA polymerase 2×Phanta Max Kit (CAT # P515, Vazyme, Nanjing, China).

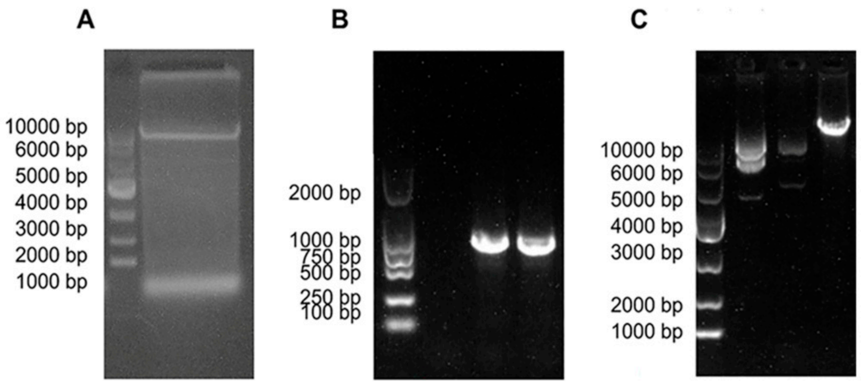

Figure S2 Cloning of the *gul* gene cluster. (A) Amplification of the *gul* gene cluster. From left to right, DNA marker and the PCR fragment of *gul* gene cluster. **(B) Screening of vector pSAT-GUL.** From left to right, DNA marker, PCR fragments with primers gul-cF and gul-cR from blank control and two positive pSAT-GUL colonies. **(C) Plasmids.** From left to right, DNA marker, plasmid pSAT209, pSET152 and pSAT-GUL.

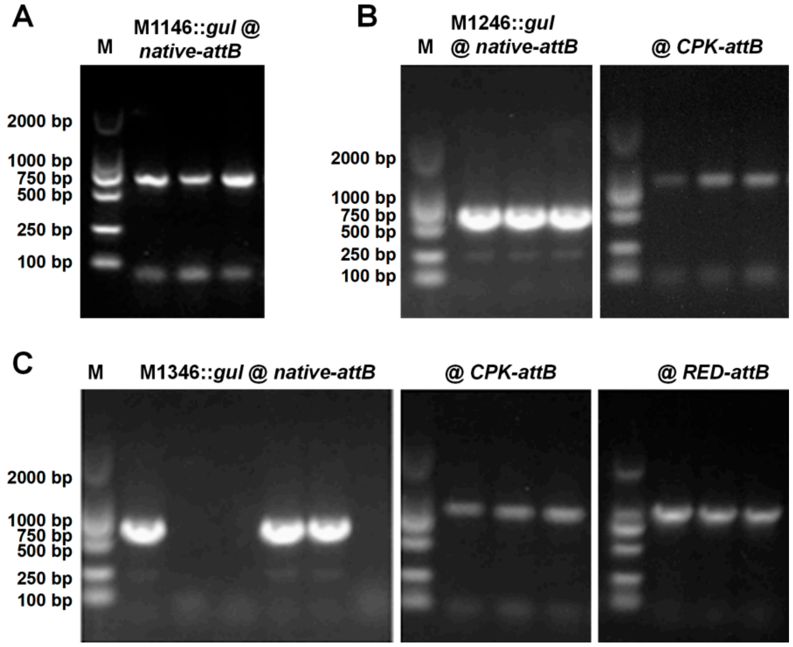

Figure S3. PCR validation of *gul* cluster integration in the *attB* loci. A. Exconjugants were checked for M1146::*gu*l integration at the native *attB* locus. Positive integration resulted in a 557 bp band. **B. Exconjugants were checked for M1246::2*gu*l integration at the native and *CPK attB* loci.** Positive integration resulted in a 557 bp and a 773 bp band, respectively. **C. Exconjugants were checked for M1346::3*gul* integration at the native, *CPK* and *RED attB* loci.** Positive integration resulted in a 557 bp, a 773 bp and an 887 bp band, respectively.

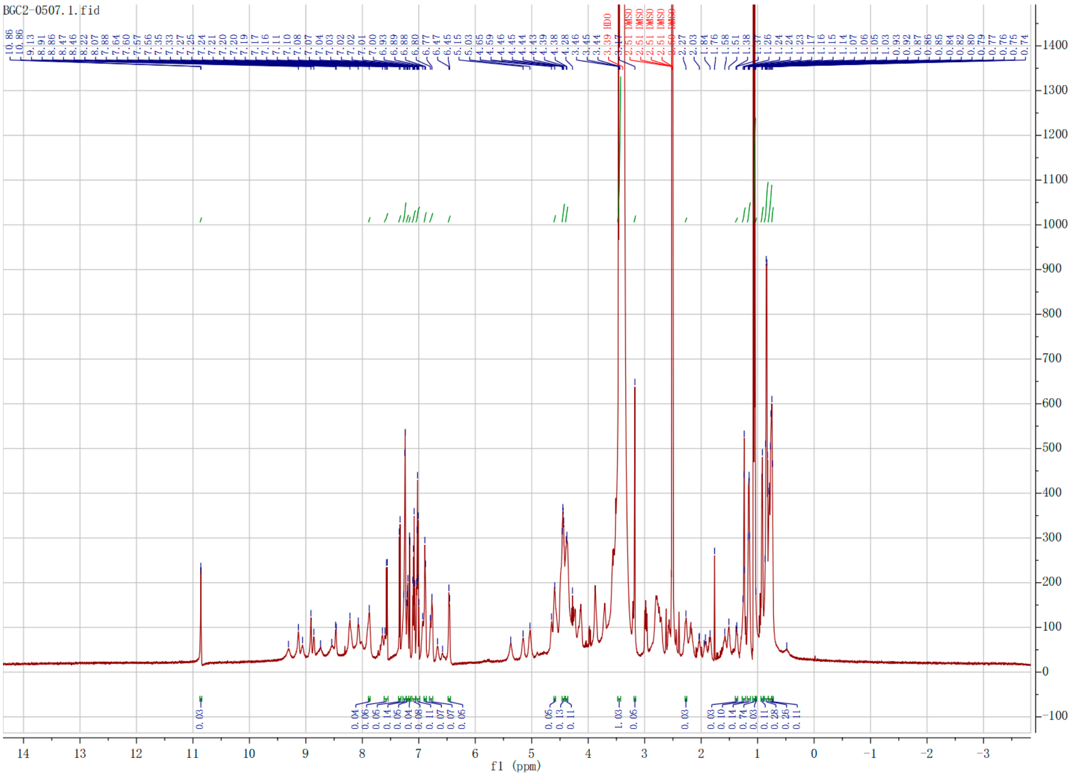

Figure S4. ¹H NMR (600 MHz) spectrum of aborycin in DMSO.

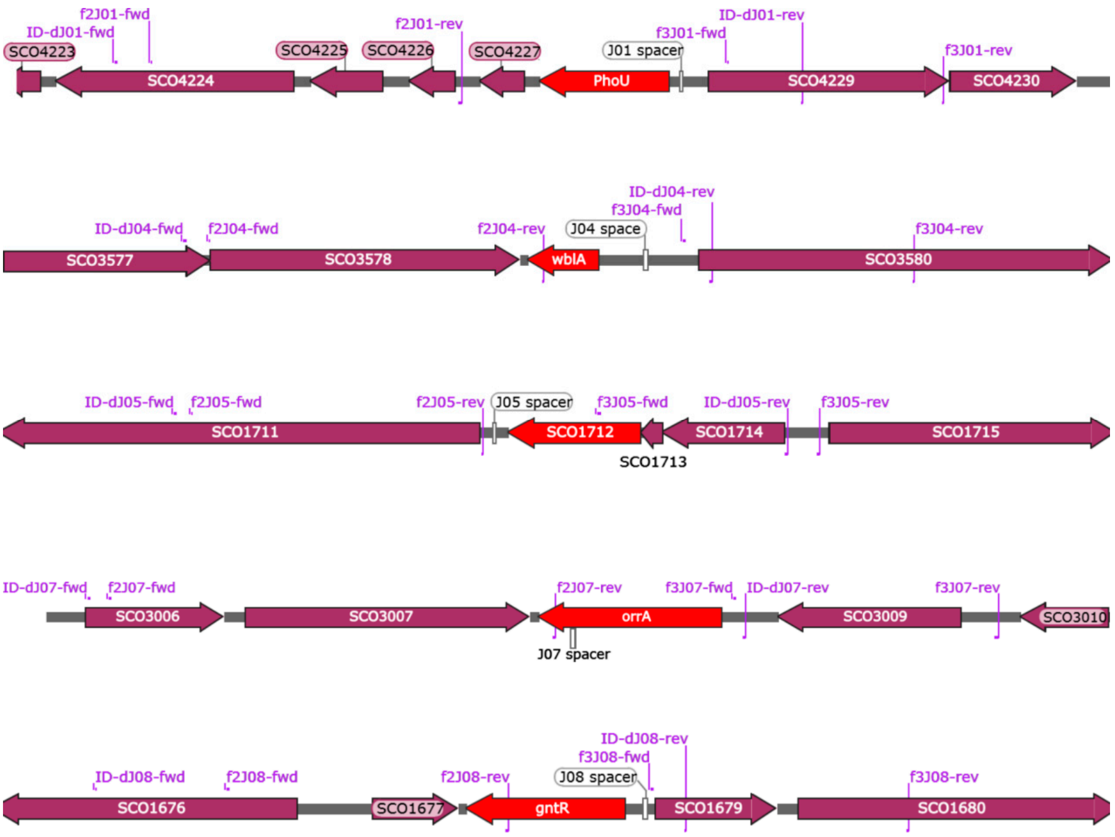

Figure S5 CRISPR/Cas9 constructions of the five gene-knockout mutants from M1346::3*gul*. Gene organizations, spacer positions and primers for deletion of *phoU*, *wblA*, *SCO1712*, *orrA* and *gntR* genes, respectively.

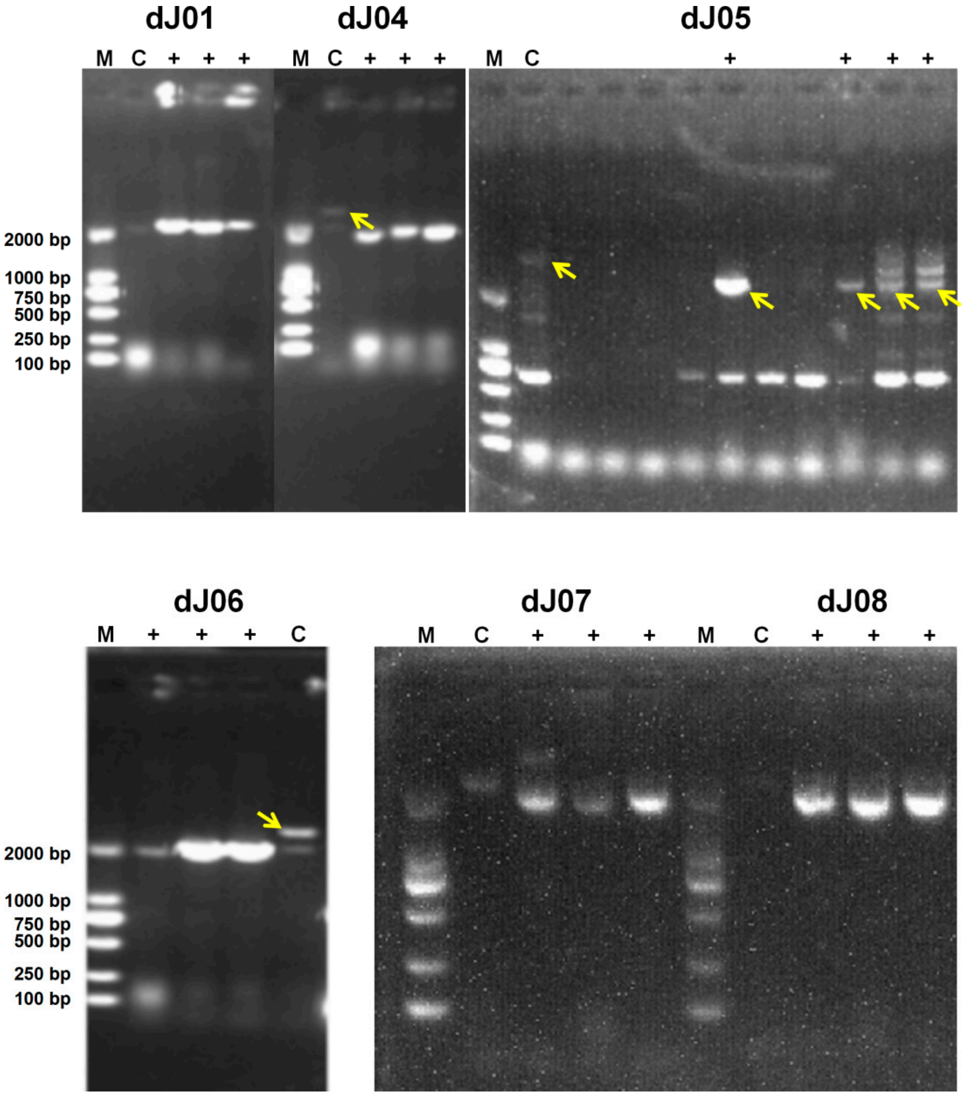

Figure S6 PCR screening for positive mutants. d01 - d08 shows screening for *ΔphoU*, *ΔwblA*, *ΔSCO1712*, *ΔorrA* and *ΔgntR* mutants of M1346::3*gul*, respectively. C, the control strain M1346::3*gul*. M, DNA marker. +, positive exconjugant. Yellow arrow, to indicate the correct band. All correct bands from the positive exconjugants were sequenced to confirm the success of deletion.

Table S1 Information of plasmids and strains

Plasmids	Relevant features	Source/Reference
pGM1190	*tsr*, rep^{pSG5}, *oriT*, *acc(3)IV*, ori^{pUC}	[46]
pSET152	*oriT*, $attP_{\Phi C31}$, $int_{\Phi C31}$, *lacZα*, ori^{pUC}, *acc(3)IV*	[43]
pUC57	*bla*, ori^{pUC}, *lacZα*	Thermo Scientific
pSAT209	*tsr*, *oriT*, $attP_{\Phi C31}$, $int_{\Phi C31}$, *bla*, ori^{pUC}	This study
pSAT-GUL	*tsr*, *oriT*, $attP_{\Phi C31}$, $int_{\Phi C31}$, *bla*, ori^{pUC}, *gul*	This study
pKCcas9	*acc(3)IV*, ori^{pUC}, rep^{pSG5}, *oriT*, *tipA-Scocas9*	[24]
pKCcas9dO	*acc(3)IV*, ori^{pUC}, rep^{pSG5}, *oriT*, *tipA-Scocas9*, j23119-*actII-orf4*-gRNA, homologous region flanking *actII-orf4*	[24]
pKCCas9-dJ01	*acc(3)IV*, ori^{pUC}, rep^{pSG5}, *oriT*, *tipA-Scocas9*, j23119-J01spacer-gRNA, homologous region flanking *phoU*	This study
pKCCas9-dJ04	*acc(3)IV*, ori^{pUC}, rep^{pSG5}, *oriT*, *tipA-Scocas9*, j23119-J04spacer-gRNA, homologous region flanking *wblA*	This study
pKCCas9-dJ05	*acc(3)IV*, ori^{pUC}, rep^{pSG5}, *oriT*, *tipA-Scocas9*, j23119-J05spacer-gRNA, homologous region flanking *SCO1712*	This study
pKCCas9-dJ07	*acc(3)IV*, ori^{pUC}, rep^{pSG5}, *oriT*, *tipA-Scocas9*, j23119-J07spacer-gRNA, homologous region flanking *orrA*	This study
pKCCas9-dJ08	*acc(3)IV*, ori^{pUC}, rep^{pSG5}, *oriT*, *tipA-Scocas9*, j23119-J08spacer-gRNA, homologous region flanking *gntR*	This study
**Strains**	**Relevant features**	**Source/Reference**
E.coli ET12567(pUZ8002)	*dam*, *dcm*, *hsdS*, *cat*, *tet*, *tra*, *neo*, RP4	[47]
Streptomyces sp. HNS054	Source strain of the aborycin gene cluster *glu*	[5]
S.coelicolor M1146	*S. coelicolor* M145 (contains one native $attB_{\Phi C31}$) *Δact*, *Δred*, *Δcpk*, *Δcda*	[48]

S.coelicolor M1246	*S. coelicolor* M1146 *Δcpk*::*attB*$_{\Phi C31}$	[25]
S.coelicolor M1346	*S. coelicolor* M1246 *Δred*::*attB*$_{\Phi C31}$	
S.coelicolor M1446	*S.coelicolor* M1346 *Δcda*::*attB*$_{\Phi C31}$	
S.coelicolor M1546	*S.coelicolor* M1446 *Δact*::*attB*$_{\Phi C31}$	
M1146:: pSAT209	*S.coelicolor* M1146 integrates one copy of pSAT209 at the native *attB*$_{\Phi C31}$	This study
M1146::*gul*	*S.coelicolor* M1146 integrates one copy of pSAT-GUL at the native *attB*$_{\Phi C31}$	This study
M1246::2*gul*	*S.coelicolor* M1246 integrates two copy of pSAT-GUL at two *attB*$_{\Phi C31}$ sites	This study
M1346::3*gul*	*S.coelicolor* M1346 integrates three copy of pSAT-GUL at three *attB*$_{\Phi C31}$ sites	This study
M1346::3*gul ΔphoU*	*S.coelicolor* M1346::3*gul* without the *phoU* gene	This study
M1346::3*gul ΔwblA*	*S.coelicolor* M1346::3*gul* without the *wblA* gene	This study
M1346::3*gul* ***ΔSCO1712***	*S.coelicolor* M1346::3*gul* without the *SCO1712* gene	This study
M1346::3*gul* ***ΔorrA***	*S.coelicolor* M1346::3*gul* without the *orrA* gene	This study
M1346::3*gul* ***ΔgntR***	*S.coelicolor* M1346::3*gul* without the *gntR* gene	This study

Table S2 Primer sequence used in this study

Primers	Sequence (5'-3')
054lasF	cgattcgtcagtgatgatatcTCCGTTGGCAAGGTTTGATGTG
054lasR	aaggatccactagtgatatcACGACGAGAAGGAGACCGAGGC
f1pUCfw	gaaatggACCGTCATCACCGAAAC
f1pUCrev	gccaaccaATAACGCAGGAAAGAACAT
f2pSETfw	gcgttatTGGTTGGCTTGGTTTCAT
f2pSETrev	ccgctctCACGCCCTCCTACATCG
f3TSRfw	agggcgtgAGAGCGGGGAGCTTTG
f3TSRrev	atgacggtCCATTTCTGGGTCCTGTTG
gul-cF	TGTCTATCGCTCCTTCGTTTCCA
gul-cR	GTCTCCCTGCTCTTCACCTCGTT
ID-oriT-fw	gcagagcaggattcccgttgagca
ID-native-attB-rev	acgtcccgtgctcaccgtgacca

ID-CPK-attB-rev		cccatgctgtgcccgaagaa
ID-RED-attB-rev		tccagcgcttggtggcgtacagctt
ID-CDA-attB-rev		catgacgcaacgcgaagaagagct
ID-ACT-attB-rev		tggtcaccgtctccatggtcatgt
f1gRNA-R		gtggaattcagatctcaaaaa
J01 set	f1J01-fwd	agtcctaggtataatactagtGCCCCCACGCTTACACGTTCgttttagagctagaaatagca
	f2J01-fwd	tttttgagatctgaattccacAGGAACTGCGGGGTGGA
	f2J01-rev	CTGGAAGGAGGACCCTGAAG
	f3J01-fwd	cttcagggtcctccttccagGAGCGGGAGCAGAAGCG
	f3J01-rev	acgacggccagtgccaagcttCGAACGGGGCTGTATGGG
	ID-dJ01-fwd	ccgttgttgtcccagaagc
	ID-dJ01-rev	cagctcgtggctcacgttg
J04 set	f1J04-fwd	agtcctaggtataatactagtTCATCTCTGTTGCGCCACAAgttttagagctagaaatagca
	f2J04-fwd	tttttgagatctgaattccacTGCGGAAGCAGGGGATC
	f2J04-rev	GCGGACGGAGTACGAACG
	f3J04-fwd	CTCGTTCGTACTCCGTCCGCTCACTGCTGTGACAGTTGAGTGC
	f3J04-rev	acgacggccagtgccaagcttAGCGTCGTGCGGATGG
	ID-dJ04-fwd	CGCTGGAGCAGGAGCAG
	ID-dJ04-rev	TGACACCGAGGAACTTGGC
J05 set	f1J05-fwd	agtcctaggtataatactagtCGGTAGGCATGTTGGGGAGGgttttagagctagaaatagca
	f2J05-fwd	gaattccacGTGTTGAAGGCGAAGTCGTGG
	f2J05-rev	aacaagcgccagatggTTCCGGGAGGACCCGTGC
	f3J05-fwd	ggaaCCATCTGGCGCTTGTTGC
	f3J05-rev	acgacggccagtgccaagcttCGTAGGATTGCGGTTTCCC
	ID-dJ05-fwd	ATCTCCGTCTCGGCCATCCTG
	ID-dJ05-rev	CCCGTGTTCTAGTCTCGCGTCAT
J07 set	f1J07-fwd	agtcctaggtataatactagtGTCCCGGTTGTTCATTCCCGgttttagagctagaaatagca
	f2J07-fwd	tttttgagatctgaattccacGGACCCCGACATCCAACG
	f2J07-rev	GGAGAAACTCCAGCTGCACTC
	f3J07-fwd	gagtgcagctggagtttctcCGCCAACCCAGAGCAACGA
	f3J07-rev	acgacggccagtgccaagcttGCGAGATATGTTCGGTTGTGAG
	ID-dJ07-fwd	AACCCGTCACGCTCACC
	ID-dJ07-rev	GGGCATGGAATCATGGC
J08 set	f1J08-fwd	agtcctaggtataatactagtGTTTCAGTTGCGTCGACATCgttttagagctagaaatagca
	f2J08-fwd	tttttgagatctgaattccacGTGGGTCCAGTCGTCCG
	f2J08-rev	GTCGTCGCCGAGGTCC

	f3J08-fwd	gccaggacctcggcgacgacCGACGAAGACAGCAGACAGC
	f3J08-rev	acgacggccagtgccaagcttCCGCATCAGCGAGTAGTTG
	ID-dJ08-fwd	GGTGTTGCTCAGGGTGGC
	ID-dJ08-rev	CGGAGGGTGGAAGTCGTC

Table S3 The length of RiPP biosynthetic gene clusters

Class/example	Length	Reference
Proteusins	13.6kb	[9]
Lasso peptides		[49]
MS-271 (siamycin)	14kb	
specialicin	14kb	
Thiopeptides		[50]
thiomuracin	13.4kb	
berninamycin	11kb	
lactazole	8.4kb	
Linaridins		[51]
cypemycin	8.5kb	
legonaridin	8.5kb	
Cyanobactins	<10kb	[52]
Bottromycins	17kb	[53]
Microviridin	6.8kb	[54]
Glycocins	4kb	[55]
Microcins		[56]
Class I	4-5.5kb	
Class II	3.5-4kb	
Class III	8.5-11.5kb	

Table S4 RiPP examples reported by Montalbán-López et al. [32]

Class/example	length
Crocagins/Crocagin A	13kb
Sulfatyrotides/RaxX	4kb
Darobactin	7kb
Tryptorubin A	4kb
Cacaoidin	28kb
Cittilins/Cittilin A	1.9kb
Lipolanthines/Microvionin	23kb
Thiopeptide/Thiomuracin	13kb

Linaridin/Cypemycin	13kb
LAP /Microcin B17	6kb
Thioamitide/Thioholgamide	10kb
Thioamitides/Thioviridamide	11kb
Cyanobactin /Patellamide	13kb
Bottromycins /Bottromycin A1	18kb
Lasso peptide/Microcin J25	6kb
Sactipeptide/Subtilosin A	8kb
Ranthipeptide/Freyrasin	4kb
Streptide /Streptide	4kb
Epipeptides/Yyd	5kb
Spliceotides/Plp	12kb
Graspetide/Microviridin J	3kb
Dikaritins/Ustiloxin	20kb
Asperipin 2a	5kb
Phomopsins	20kb
Pheganomycin	28kb
Pyrroloquinoline quinones/PQQ	9kb
Mycofactocin/Mycofactocin	15kb
Methanobactins/Methanobactin	13kb
3-thiaglutamate	15kb
Aeruginosamide B	15kb
Landornamide	14kb
Goadsporin	23kb
Thiocillin	16kb
